# Retraction: The influence of the ultrasonic treatment of working fluids on electrospun amorphous solid dispersions

**DOI:** 10.3389/fmolb.2025.1734145

**Published:** 2025-11-07

**Authors:** Frontiers Editorial Office

The journal retracts the 10 May 2023 article cited above.

Following publication, concerns were raised regarding the integrity of the images in the published figures. Specifically, image duplication concerns were identified in Figure 4 which was found to contain an unacceptable level of similarity to Figure 5 in a previously published article by Li, X., Yu, D., Fu, C., Wang, R. and Wang, X. (2013) Ketoprofen/ethyl Cellulose Nanofibers Fabricated Using an Epoxy-coated Spinneret. Modeling and Numerical Simulation of Material Science, 3, 6-10. doi: 10.4236/mnsms.2013.34B002

The authors failed to provide a satisfactory explanation during the investigation, which was conducted in accordance with Frontiers’ policies. As a result, the data and conclusions of the article have been deemed unreliable and the article has been retracted.

This retraction was approved by the Chief Editors of Frontiers in Molecular Biosciences and the Chief Executive Editor of Frontiers. Frontiers would like to thank Fabian Wittmers for contacting the journal regarding the published article.

